# Effects of silver impurity on the structural, electrical, and optical properties of ZnO nanowires

**DOI:** 10.1186/1556-276X-6-552

**Published:** 2011-10-10

**Authors:** Kyoungwon Kim, Pulak Chandra Debnath, Deuk-Hee Lee, Sangsig Kim, Sang Yeol Lee

**Affiliations:** 1 Electronic Materials Center, Korea Institute of Science and Technology, Seoul 136-791, Korea; 2 Department of Electrical Engineering and Institute for Nano Science, Korea University, Seoul 136-701, Korea; 3 Department of Nanoelectronics, School of Engineering, University of Science and Technology, 52 Eoeun dong, Yuseong-gu, Daejeon 305-333, Republic of Korea; 4 Department of Semiconductor Engineering, Cheongju University, Cheongju, 360-764, Chungbuk, Korea

## Abstract

1, 3, and 5 wt.% silver-doped ZnO (SZO) nanowires (NWs) are grown by hot-walled pulsed laser deposition. After silver-doping process, SZO NWs show some change behaviors, including structural, electrical, and optical properties. In case of structural property, the primary growth plane of SZO NWs is switched from (002) to (103) plane, and the electrical properties of SZO NWs are variously measured to be about 4.26 × 10^6^, 1.34 × 10^6^, and 3.04 × 10^5 ^Ω for 1, 3, and 5 SZO NWs, respectively. In other words, the electrical properties of SZO NWs depend on different Ag ratios resulting in controlling the carrier concentration. Finally, the optical properties of SZO NWs are investigated to confirm *p*-type semiconductor by observing the exciton bound to a neutral acceptor (A^0^X). Also, Ag presence in ZnO NWs is directly detected by both X-ray photoelectron spectroscopy and energy dispersive spectroscopy. These results imply that Ag doping facilitates the possibility of changing the properties in ZnO NWs by the atomic substitution of Ag with Zn in the lattice.

## 1. Introduction

As an important II-VI semiconductor, ZnO is a promising material, for the use in ultraviolet or visible optoelectronic device, because of its large exciton binding energy (60 meV) and direct wide band gap (3.37 eV) [1-4]. For decade, ZnO NWs have attracted a considerable amount of research interest because of the potential applications for nano-scale optoelectronic devices, such as light emitting diodes (LED), field effect transistors (FETs), solar cells, and ultraviolet (UV) lasers [5-7]. Compared with thin film structures, one-dimensional (1D) semiconductor devices, such as nanotube [8], nanoribbons [9,10], and nanowires (NWs) [11-13], could enable high efficiency, enhanced performance, new functions, and diverse applications [14-17]. 1D materials have received great attention because of their much potential for fundamental studies of the roles of dimensionality and size on their properties, as well as for their applications in nano-devices [18]. The success of nano-devices similarly trusts on the ability of controlling the transport and electrical properties of the semiconductors, such as in thin film doping techniques. Doping by introducing electron donor or acceptor elements into the host crystal is a successful approach in thin film or thick film devices. However, such doping approach remains a challenge for 1D nano-device semiconductors [16]. Normally, ZnO exhibits *n*-type conductivity because of native defects, such as oxygen vacancies and zinc interstitials. The strong *n*-type conductivity of ZnO restricts the application and it is difficult to fabricate *p*-type conductive ZnO [19], and the realization of *p*-type ZnO is rather difficult because of its asymmetric doping limitations [20]. Recently, research of ZnO has been focused on the synthesis of *p*-type ZnO using various dopants, such as N, P, As, Sb, and Ag [18,21-23]. Among possible acceptor dopants, silver (a group Ib element) is a good candidate for producing a shallow acceptor level in ZnO, if incorporated on substituted Zn sites [24]. However, there has been no report on the fabrication of *p*-type ZnO nano-structures by Ag dopant. The Ag-doped ZnO thin films for the various applications have been reported by Kang et al. [25]. They demonstrated that the Ag ion can be substituted into the site of Zn ion and a narrow processing window region exists to fabricate the *p*-type ZnO using Ag as *p*-type dopant source [25]. *p*-Type doping effect is confirmed by low temperature photoluminescence (PL) spectroscopy that is a very sensitive tool for the characterization of acceptor/donor impurities and is helpful in understanding the optical and electrical performances of the materials. We focused on the temperature-dependent PL measurements of various silver-doped ZnO (SZO) NWs to reveal the role of Ag acceptor in the optical properties of the ZnO-based NWs.

## 2. Experiment procedure

Compared with chemical vapor deposition methods, physical vapor deposition (PVD) guarantees a cost-effective process as well as easy energy control because of the relatively simple design and operation principle [26]. Based on the vapor-liquid-solid (VLS) mechanism in the PVD method, various SZO NWs have been synthesized on (0001) sapphire substrates in hot-walled pulsed laser deposition (HW-PLD) with 20 Å Au film as a catalyst. ZnO targets doped with Ag_2_O (1, 3, and 5 wt.%) made from pressed (1600 kg/cm^2 ^in the cold isostatic pressing) and sintered (950°C for 3 h) high purity powders (Kojundo, 99.999%) were adopted. The Al_2_O_3 _substrates were cleaned in acetone and methanol for 20 min and rinsed in de-ionized water for 5 min. The 1, 3, and 5 wt.% SZO (5SZO) NWs are grown in a furnace at 800°C with argon gas of 90 sccm and a working pressure of 1.2 torr. The *c*-plain sapphire substrate was set at a fixed distance (2.5 cm) downstream the particles as a collection substrate. The HW-PLD has a target rotating system ensuring homogeneous target ablation. A KrF excimer laser with the wavelength of 248 nm operating at a pulse repetition rate of 10 Hz was focused onto 1, 3, and 5SZO targets for the deposition. The laser influence was set to 1.2 J/cm^2^, and the shot area on the target surface was 0.042 cm^2^. Before the deposition, there should be pre-deposition process for 5 min. The deposition process continued for 30 min. The structural and optical properties of the nanostructures were investigated by field emission scanning electron microscopy (FE-SEM), transmission electron microscopy (TEM), and low temperature PL, respectively. Ag element is observed in ZnO NWs using both X-ray photoelectron spectroscopy (XPS) and energy dispersive spectroscopy (EDS), which are carried out to investigate the elemental composition of ZnO-based NWs

## 3. Results and discussion

**Figure 1:**
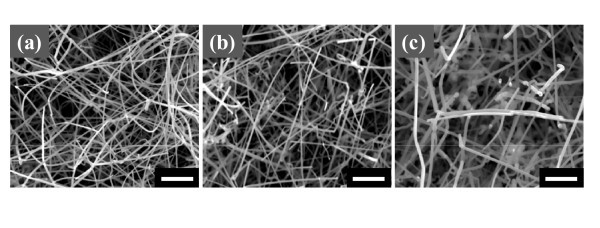
**Top-view FE-SEM images of various SZO NW**. **(a) **1SZO, **(b) **3SZO, and **(c) **5SZO NWs are fabricated by using 1, 3, and 5 wt.% Ag-doped ZnO ceramic targets. The scale bar is 1 μm.

**Figure 2:**
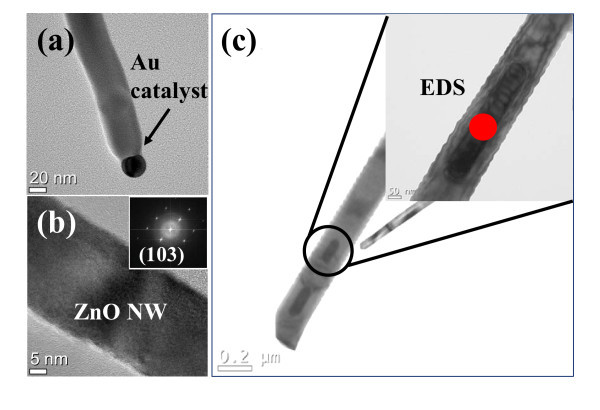
**TEM images of 3SZO NW**. **(a) **The location and the size of the Au catalyst, the present of Au catalyst, is demonstrated the VLS method. **(b) **The HR-TEM and SAED pattern (inset) reveal the (103) primary growth plane of the 3SZO NW. **(c) **TEM image of Ag cluster shape in the 3SZO NWs. The inset is a HR-TEM image of the 3SZO NWs. The metal cluster, red circle area, is measured Ag concentration ratio of 36.79 wt.% using TEM-EDS.

**Figure 3:**
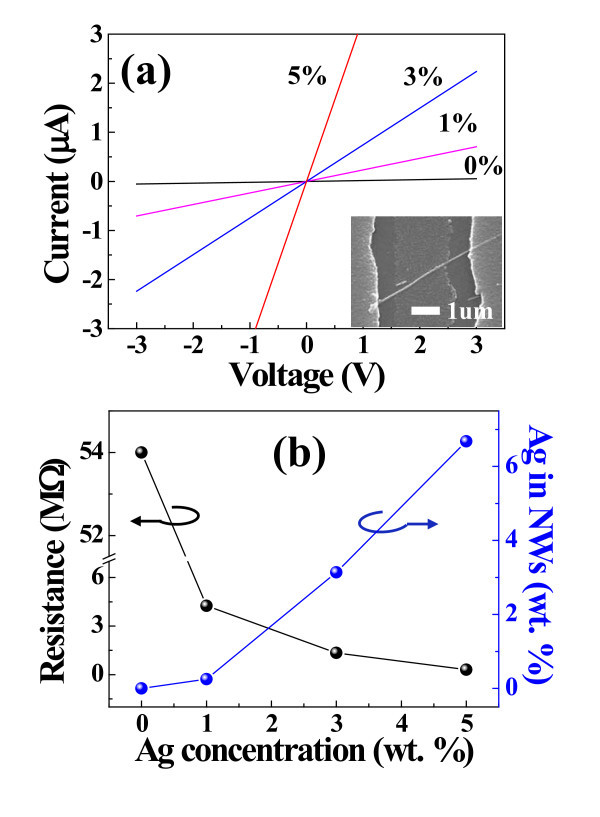
**Physical properties of varios SZO NW**. **(a) **Tuned resistances of un-doped, 1SZO, 3SZO, and 5SZO NWs, the resistances of the SZO NW are measured to be about 54.1, 4.255, 1.34, and 0.304 MΩ, respectively. The inset is the SEM image of the SZO NW device. **(b) **Quantitative analysis of EDS is measured at 0, 0.25, 3.14, and 6.68 wt.% for the un-doped, 1SZO, 3SZO, and 5SZO, respectively.

The optimized growth condition for the various SZO NWs is accomplished by self-designed HW-PLD. Figure 1 shows the top-view FE-SEM images of various SZO NWs grown using 1, 3, and 5 wt.% Ag-doped ZnO ceramic targets. As shown in Figure 1a, b, the distribution of the SZO NWs is random, with the average diameter of about 60 nm and the average length of about 8 μm. However, irregular distribution of the SZO NWs with different shapes has been observed by increasing Ag doping concentration. 5SZO NWs are observed notable non-uniformity in shape, diameter, and length. It is considered that the irregularity of the heavily doped NW stems from the lattice stress induced by the substitution of Ag with Zn [26]. Figure 2 shows TEM images of 3 wt.% SZO (3SZO) NW. The selected-area electron diffraction (SAED) pattern (inset in Figure 2b) reveals the (103) primary growth plane of 3SZO NW. It confirms significantly that the primary growth plane of the NWs was switched from (002) plane to (103) plane by introducing Ag into the ZnO lattice. Many researchers reported primary growth direction of the ZnO NW by high-resolution TEM, SAED pattern, and X-ray diffraction method [4,19,25]. The ZnO NW grown on *c*-axis sapphire substrates generally has (002) primary growth direction, in accordance with the lowest (001) surface energy and small lattice mismatch of hexagonal ZnO structures [27]. However, it has been reported that Ag-doping induced a transition from (002) plane to both (101) and (103) planes caused by Ag-doping effect [26]. The primary growth (103) plane of SZO NWs explains to reduce doping stress in the ZnO lattice because the Ag^+ ^ions have a larger radius (0.122 nm) compared with the host Zn^2+ ^ions (0.072 nm). Also, we can verify the location and the size of the Au catalyst in formed SZO NWs as shown in Figure 2a. The Au catalyst has been observed at the top of NW with compatible size of NW diameter, which indicates the VLS method to make SZO NWs structure [28]. Figure 2c is the TEM image of Ag cluster shape in the 3SZO NWs. After Ag-doping process, Ag clusters are observed in ZnO NW, which can increase conductance of SZO NWs, as shown in Figure 3.

To investigate the change of SZO NW resistance, resistances of various SZO NWs are measured using two-probe method with the Ti/Au electrodes on both sides of the single SZO NW. Linear *I*-*V *curves are obtained, indicating ohmic contacts between SZO NW and Ti/Au electrodes. Figure 3 shows tuned resistances of un-doped, 1, 3, and 5SZO ZnO NWs to be about 54.1, 4.255, 1.34 and 0.304 MΩ, respectively. Based on theory, the role of Ag dopants is to reduce majority carriers (as electrons) in the ZnO matrix, when Ag ion is substitute in Zn site. Therefore, the minority carriers (as holes) of SZO NW are increased because Ag element is I group. So, the resistance of SZO NW is continuously increased as the concentration of Ag element increased. Finally, the resistance of SZO NW is decreased oppositely when the number of the minority carriers (as hole) is higher than the number of majority carriers (as electron). Therefore, SZO NW FETs have to show *p*-type behavior.

However, our SZO NW FET does not show *p*-type because working temperature is too high. Instead of *p*-type behavior, our SZO NW FET shows properties of *n*-type and continuously increasing conductivity. At high working temperature, Au catalyst and Ag dopant are combined as liquid state at the top-end of nanowire because nanowire is grown by VLS method. We already confirmed the ratio of Au catalyst and Ag dopant by TEM-EDS. These combined metals continuously become bigger caused by added Ag dopants. These excess Ag dopants are existed as Ag metal cluster in the SZO NW, which acts as electron path, and this increase the mobility of SZO NW FET. So, the conductivity of SZO NW is continuously increased according to increasing Ag dopants. The inset is the SEM image of the SZO NW device. These results show that the electrical properties of SZO NWs are changed with different Ag ratios caused by controlling the carrier concentration by doping Ag into ZnO NWs. Recently, Kim et al. [29] reported that the effect of Ag dopant on the ZnO-based FET improved electrical properties caused by increased mobility. The EDS analysis reveals the presence of Ag, Zn, and O elements. Quantitative analysis of EDS reveals that Ag concentrations are proportional to those of the targets, and measured to be about 0.25, 3.14, and 6.68 wt.% for the 1 wt.% SZO (1SZO), 3SZO, and 5SZO, respectively, as shown in Figure 3b. The PLD has several notable advantages. One of the advantages is very effective in obtaining stoichiometry-synthesized materials on the substrate same as a target than many other gas-surface-based growth techniques [30]. The HW-PLD enables the synthesis of oxide NWs while controlling the doping concentration feature. The doping could be controlled by adjusting the target composition since it guarantees the transfer of the composition from the target to the NWs [28]. Figure 3b shows that the weight ratio of various SZO targets is transformed to each SZO NWs, and demonstrate that the doping control of ZnO NWs is possible by the concentration control of Ag wt.% in targets.

**Figure 4:**
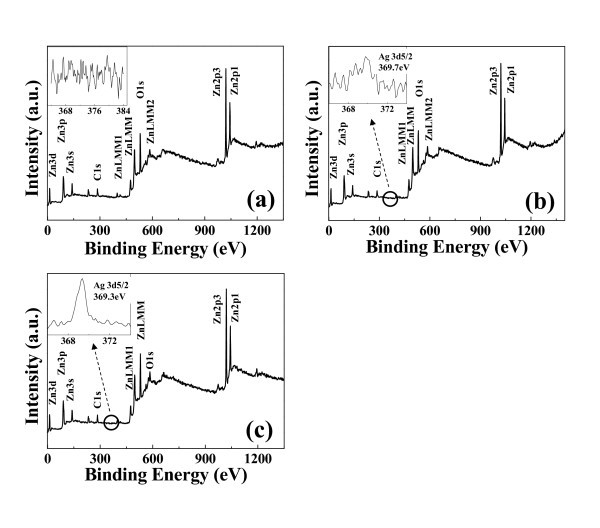
**XPS results of SZO NW**. The origin of the chemical bonding and content of Ag element are measured by XPS measurement for the **(a) **1SZO, **(b) **3SZO, and **(c) **5SZO NWs. The insets show that the shape strong peaks originated from the Ag chemical bounding peak (Ag 3d_5/2_) of the SZO NWs are observed at 369.7 and 369.3 for 3SZO and 5SZO NWs, respectively.

To understand the origin of the chemical bonding, binding energy of Ag element is investigated using XPS measurement for the Ag-doped with 0.23, 3.14, and 6.68 wt.% SZO NWs, which is confirmed by EDS data, as shown in Figure 4. The 3SZO and 5SZO NWs show Zn, O, and Ag orbital in Figure 4b, c. However, in case of 1SZO NW has not been observed mainly Ag orbital because it is very hard to detect a little quantity by XPS. Similarly, Yuan et al. [16] reported that XPS data did not show Ga element less than 0.2 at.%. A sharp strong peak originated from Ag chemical bounding peak (Ag 3d_5/2_) of the SZO NWs is observed at 369.7 and 369.3 eV for 3SZO and 5SZO NWs, respectively, as shown in the inset of Figure 4b, c. Also, both 369.7 and 369.3 eV are close to the binding energy of Ag 3d_5/2 _of Ag-O bond. In the case of 5SZO NWs with 6.68 wt.% Ag quantity, it shows very sharp and high intensity according to high amounts of Ag concentration, indicating the successful Ag-doping into the ZnO structure.

**Figure 5:**
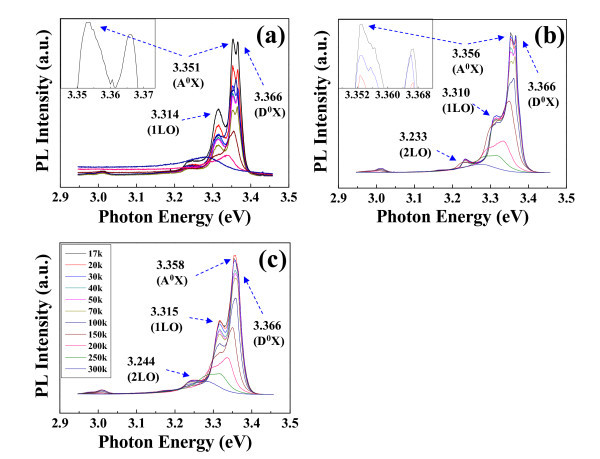
**Temperature-dependent PL spectrums from (a) 1SZO, (b) 3SZO, and (c) 5SZO NWs**. A sharp strong peak originated from the NBE (the exciton bound to neutral acceptor) of the ZnO-based NWs was observed at about 3.351, 3.356, and 3.358 eV, respectively.

Figure 5 shows PL spectrums of the various SZO NWs depending on temperature. Temperature is increased from 17 K to room temperature (RT) to detect the exciton peak that has been screened by the phonon vibration at elevated temperatures. Sharp strong peaks originated from the near band-edge emission (NBE) of ZnO-based NWs are observed at around 3.351, 3.356, and 3.358 eV. The temperature-dependent PL of the SZO provides the reference for the PL analysis of the doped ZnO NWs, in which dominant peaks of A^0^X are clearly observed at 3.351, 3.356, and 3.358 eV for 1SZO, 3SZO, and 5SZO, respectively, as shown in Figure 5[31]. It demonstrates that Ag ion is successfully substituted into the site of Zn ion, and Ag dopant can act as a desirable acceptor in ZnO NWs. It is very interesting to note that the low-temperature PL of various SZO NWs has two kinds of peaks, such as A^0^X and exciton bound to a neutral donor (D^0^X) [26,32]. It indicates that SZO NWs included optically *p*-type semiconductor. Especially, in case of A^0^X peak of 5SZO NWs is shown as very strong compared with D^0^X peak, therefore, Ag dopants in 5SZO NW strongly act the majority carriers caused by much Ag quantity of 6.68 wt.%, which is confirmed by EDS spectrum. As the temperature decreased, the blue shift of the peak to a shorter wavelength was observed, because of the band gap broadening effect at low temperatures which has been reported earlier [33]. Other peaks of the SZO NWs are observed at about 3.310 and 3.233 eV that are disappeared with phonon vibration at elevated temperatures, as shown in Figure 5. The peaks, originating from the longitudinal optical (LO) phonon replica emission in the ZnO-based materials, are also clearly observed. As depicted in the references, it is verified that the 2LO phonon replica peak is about 77 meV apart from 1LO peak [33]. With the low-temperature PL analysis, we can conclude that Ag-doping facilitates optically *p*-type in ZnO by the atomic substitution of Ag with Zn in the lattice and Ag (a group Ib element) is a good candidate to generate a shallow acceptor level in ZnO.

**Figure 6:**
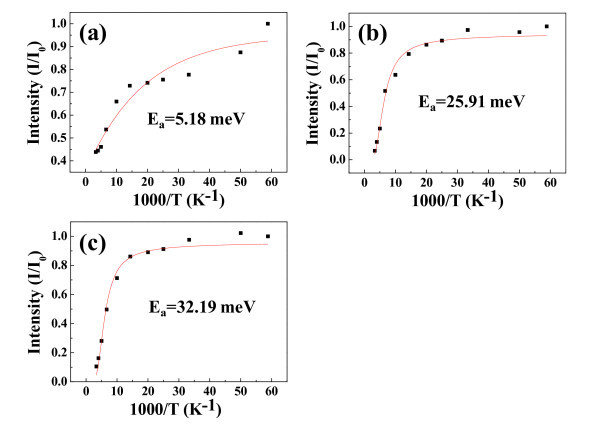
**The activation energies of the A^0^X peaks**. Arrhenius plots of exciton bound to neutral acceptor of PL emissions depending on thermal quenching from **(a) **1SZO, **(b) **3SZO, and **(c) **5SZO NWs. The activation energies of the A^0^X formation are calculated to be about 5.18, 25.91, and 32.19 meV for 1SZO, 3SZO, and 5SZO NWs, respectively.

To investigate the influence of Ag doping into SZO NWs with different Ag quantity, we have derived the Arrhenius plots of A^0^X peaks by PL emissions depending on thermal quenching for the SZO NWs, as shown in Figure 6. The Arrhenius equation gives the quantitative basis of the relationship between the activation energy (*E*_a_) and the rate at which a reaction proceeds. From the Arrhenius equation, the *E*_a _can be expressed as [34]

(1) $$I=I_{0}∕\left[1+A\;exp\left(-E_{a}∕kT\right)\right]$$

where *E*_a _is the activation energy of PL emission for thermal quenching, *I*_0 _and *A *are scaling factors, and *k *is Boltzmann's constant. The activation energies of the A^0^X formation are calculated to be about 5.18, 25.91, and 32.19 meV for 1SZO, 3SZO, and 5SZO NWs, respectively, as shown in Figure 6. This result demonstrates that the *E*_a _of SZO NWs is very sensitive depending on the concentration of Ag elements. It is very interesting to note that the *E*_a _of 1SZO shows low value compared with both 3SZO and 5SZO NWs, as derived in Figure 6. In other words, the 1SZO NWs easily become Ag-doping in the ZnO-based structure because of both low *E*_a _and optimized quantity of substitution function of Ag^+ ^[19]. However, stress of lattices structure on both 3SZO and 5SZO NWs is very impressive because a lot of Ag interstitials and Ag substitutions are existed as Ag metal cluster in the ZnO matrix, as shown in Figure 2c. Figure 2c shows a TEM image of Ag cluster shape in the 3SZO NWs caused by Ag interstitials and Ag substitutions. The inset of Figure 2c is a HR-TEM image of the 3SZO NWs. The metal cluster, red circle area in Figure 2c, is measured to be with Ag concentration ratio of 36.79 wt.% using TEM-EDS. Effect of Ag clusters in 3SZO, 5SZO NWs decreases resistances because of Ag clusters of high electric conductivity, as shown in Figure 3. Also, Ag clusters act defect in 3SZO, 5SZO NWs; therefore, both 3SZO and 5SZO NWs have a high *E*_a _than 1SZO NWs. The Ag^+ ^ions have a larger radius (0.122 nm) than that of the host Zn^2+ ^ions (0.072 nm) or other group I elements. So, the lattice feels compressive stress because the chemical bonding distance of Ag-O is longer than that of Zn-O [35,36]. In the case of 3SZO and 5SZO NWs, the surface of the SZO NWs is crumpled because of the stress by doping effect [37]. Finally, we observed two kinds of effects, when Ag dopants are heavily doped in ZnO NW; (i) existed Ag metal cluster and (ii) changed rough surface morphology.

## 4. Conclusion

In summary, 1SZO, 3SZO, and 5SZO NWs have been synthesized on the sapphire substrate by self-designed HW-PLD with Au films. We have demonstrated Ag-doping in the ZnO-based NWs using EDS, XPS, and PL measurements. In case of Ag-doped ZnO NWs, primary growth direction of SZO NWs is changed from (002) to (103). Electrical analysis of various SZO NWs shows that the tuned resistances are from 4.255 to 0.304 MΩ using optimized Ag concentration, and SZO NWs exhibit different electrical property with different Ag ratios caused by controlling the carrier concentration. Ag-doping status is verified with low-temperature PL to find the exciton bound to natural acceptor in the all SZO NWs. It indicates that the Ag dopant can act as a desirable acceptor in ZnO NWs. The low temperature PL studies reveal that the *E*_a _of the Ag acceptor is calculated to be about 5.18, 25.91, and 32.19 meV for 1, 3, and 5SZO NWs, respectively. Especially, 1SZO NWs with low *E*_a _of 5.18 meV have a good condition for making Ag-doped ZnO NWs. These results demonstrate that the *E*_a _of SZO NWs is very sensitive depending on the concentration of Ag elements.

## Competing interests

The authors declare that they have no competing interests.

## Authors' contributions

KW conceived the study, conducted the experiments, performed characterization, analyzed the data, interpreted the results, and wrote the manuscript. PCD, DHL and SK helped in the technical support for experiments and characterization. SYL designed the experiments, supervised, and corrected the manuscript. All authors read and approved the final manuscript.
